# Acceptability and feasibility of insect consumption among pregnant women in Liberia

**DOI:** 10.1111/mcn.12990

**Published:** 2020-03-01

**Authors:** Katrina M. Coley, Joseph E. Perosky, Aloysius Nyanplu, Alphonso Kofa, Jacob P. Anankware, Cheryl A. Moyer, Jody R. Lori

**Affiliations:** ^1^ School of Nursing University of Michigan Ann Arbor Michigan; ^2^ Bong County Health Team, Bong County Liberia; ^3^ Department of Horticulture and Crop Production, School of Agriculture and Technology University of Energy and Natural Resources Sunyani Ghana; ^4^ College of Human Medicine Michigan State University East Lansing Michigan; ^5^ Departments of Learning Health Sciences and Obstetrics and Gynecology University of Michigan Medical School Ann Arbor Michigan

**Keywords:** food security, insect consumption, low‐income countries, malnutrition, maternal nutrition, pregnancy outcome

## Abstract

Maternity waiting homes (MWHs) in Liberia promote facility‐based delivery to reduce maternal mortality. However, women often must bring their own food and supplies to MWHs, which makes food insecurity a barrier to the utilisation of MWHs. Consumption of edible indigenous insects is a common practice and has notable nutritional benefits but has not been studied in Liberia as a potential solution to food insecurity at MWHs. The purpose of this study is to (a) examine the acceptability of insect consumption in the context of Liberian beliefs, (b) identify species commonly consumed by pregnant women in Liberia, and (c) examine the feasibility of harvesting insects as food and income generation for women staying at MWHs. Focus groups were conducted at 18 healthcare facilities in Liberia. Participants included chiefs, community leaders, women of reproductive age, traditional birth attendants, women staying at MWHs, and male partners. Focus group participants identified many different species of insects consumed by pregnant women in the community as well as the perceived health impacts of insect consumption. They also described their own experiences with insect hunting and consumption and the perceived marketability of insects, particularly palm weevil larvae. The results of these discussions demonstrate that insect consumption is an acceptable practice for pregnant women in rural Liberia. These findings suggest that it is feasible to further explore the use of palm weevil larvae as dietary supplementation and income generation for women staying at MWHs in Liberia.

**Key messages**

- This research establishes that insect consumption is already an acceptable practice throughout Liberia, even among pregnant women.
- Participants in this research note many species of insects that are currently eaten and marketable, but they highlight palm weevil larvae as a particularly appropriate source of dietary supplementation and income generation.
- Pilot studies are needed to demonstrate insect farming as a sustainable approach to combating food insecurity in maternity waiting homes in Liberia to promote the utilisation of these homes, which contribute to increased facility‐based delivery for pregnant women.

## 1 BACKGROUND

Within Liberia, maternity waiting homes (MWHs) provide transitional housing for pregnant women who are close to full term, to increase facility delivery for women living far from a health centre (Lori & Starke, 2012; Lori, Munro et al., 2013). Although infant mortality is decreasing in Liberia, maternal mortality remains unchanged (Central Intelligence Agency, 2018). MWHs play an important role in reducing maternal mortality by providing access to skilled birthing care at healthcare facilities for women who would otherwise give birth at home (Bekele, Dadi, & Tesfaye, 2019; Lori, Wadsworth et al., 2013). Although the implementation of MWHs is an important strategy to bring vulnerable women close to a health facility (Singh et al., 2018), barriers remain to optimising MWHs. One such barrier is food security. Several studies have identified lack of food provisions as a deterrent for women considering an MWH stay (Chibuye, Bazant, Wallon, Rao, & Fruhauf, 2018; Lori, Wadsworth, et al., 2013).

In many developing countries, food insecurity contributes to a vicious cycle of poor nutrition and severe adverse physical and mental health outcomes, ranging from minor ailments to chronic illness and death (Black et al., 2013; Hadley et al., 2008; Muller & Krawinkel, 2005). In Liberia, malnutrition is the number one factor driving death and disability (Institute for Health Metrics and Evaluation, 2016). Black et al. (2013) examined the consequences of maternal undernutrition and consequent fetal growth restriction, which they found causes 12% of child deaths, contributes to stunting of linear growth and resultant obesity, and acts as a determinant of non‐communicable diseases in adulthood. Despite increased efforts to improve food security in sub‐Saharan Africa to achieve the Millennium Development Goal of decreasing the proportion of the population suffering from undernourishment, approximately one person in every four remains undernourished (Food and Agriculture Organization of the United States, 2015).

In recent years, the consumption of indigenous insects has been gaining recognition as a safe dietary supplement, which can also serve as an income‐generating activity if the insects are commercially harvested (Anankware, Osekre, Obeng‐Ofori, & Khamala, 2016; Dewey & Adu‐Afarwuah, 2008; Dzerefos, Witkowski, & Toms, 2013; Illgner & Nel, 2000; Laar et al., 2017; Latham, 2015). Research further identifies specific species in various regions of the world that might contribute to insect consumption as an option to improve nutrition (Anankware et al., 2016; Dzerefos et al., 2013; Illgner & Nel, 2000; Laar et al., 2017; Latham, 2015; Raheem et al., 2019). However, cultural beliefs also impact the potential of insect consumption. A study in Tanzania found that men declared the longhorn grasshopper *Ruspolia differens* to be taboo for both women and children to consume (Mmari, Kinyuru, Laswai, & Okoth, 2017), demonstrating the need for greater understanding of the cultural beliefs surrounding insect consumption within a specific context.

In West Africa, palm weevil larvae are a traditional food source in some rural communities (Laar et al., 2017; Muafor, Gnetegha, LeGall, & Levang, 2015). Palm weevil larvae are often acquired when palm trees are deliberately cut down to produce palm wine from their sap (Van Itterbeeck & Van Huis, 2012). This practice is especially common during times when other game and fish are scarce, as the palm weevil larvae provide an important source of protein (Van Itterbeeck & Van Huis, 2012). A study from Payne, Scarborough, Rayner, and Nonaka (2016) concludes that the palm weevil is a healthier alternative to beef and chicken products and might provide an especially effective method for combating undernutrition. The palm weevil larva has been studied as a dietary supplement and income‐generating activity within Ghana and Cameroon (Laar et al., 2017; Muafor et al., 2015). The semi‐farming method of palm weevil larvae harvesting outlined by Muafor et al. (2015) details a sustainable, contained method of insect farming that can be practiced at any time of year, which gives an added benefit over traditional hunting practices. However, there is currently no such research outlining the additional cultural considerations necessary to implement this approach in Liberia. This study helps to fill this gap by exploring the beliefs and practices of insect consumption by rural Liberians and their experiences of the marketability of palm weevil larvae as an income‐generating activity.

This qualitative descriptive study was conducted as part of a larger research project to establish an evidence‐based approach to the use of MWHs and inform the overall strategic plan to improve maternal and neonatal health in Liberia with implications for global impact. The parent study for this project aims to provide in‐depth detail and evidence on the process of building and sustaining MWHs over time. Exploration into the use of palm weevil larvae as supplemental nutrition and income‐generating activity at MWHs will aid researchers in determining the next steps for improving food security and sustainability within MWHs.

## 2 METHODS

A descriptive study design was used to examine the acceptability of insect consumption by pregnant women in rural Liberia and determine the feasibility of insect farming as a mechanism to produce a marketable food supplement for women staying at MWHs in Liberia. Qualitative data were obtained through focus group discussions to answer the following research questions: (a) Is it acceptable for pregnant women in Liberia to consume insects? (b) Is it feasible to develop a palm weevil larvae farm as a form of dietary supplementation and a source of income generation at MWHs in rural Liberia? Prior to data collection, institutional review board approval was received from the University of Michigan Health Sciences and Behavioral Sciences Institutional Review Board and the University of Liberia Pacific Institute for Research and Evaluation.

### 2.1 Sample and setting

Researchers conducted focus groups at 18 healthcare facilities with associated MWHs throughout 9 of the 15 counties in Liberia. Focus group participants included a convenience sample of chiefs, community leaders, women of reproductive age, traditional birth attendants (TBAs), women currently staying at MWHs, and male partners from both the immediate area around the clinic and associated catchment communities. These catchment communities constitute the primary geographical area served by each clinic and affiliated MWH (Schuurman, Fiedler, Grzybowski, & Grund, 2006). Any respondents below the age of 18 and those who could not consent were excluded from this study.

### 2.2 Data collection

Healthcare facility staff and town chiefs used verbal announcements to recruit community members. Because of the potential power dynamic between healthcare providers and those who are receiving care in MWHs, focus groups were conducted by research assistants (RAs) and interpreters from Liberia who were trained in the ethical approach to research through the PEERRS (Program for Education and Evaluation in Responsible Research and Scholarship), a research ethics training programme. Focus groups were held in meeting rooms at the MWHs, and all non‐participants were asked to leave. Participants received a beverage and a snack valued at approximately $2. Prior to beginning each focus group, the RA provided an explanation of the study and obtained verbal informed consent from each participant. All participants were told that the focus groups were voluntary and that they could decline to answer any questions or complete the focus group even after giving initial consent. Focus group members were assured that participation would not affect the care they receive from the health facility. Participants' names were not recorded on the data collection tools.

The focus group guide included 12 questions about food provision in MWHs, insect consumption practices, perceptions of insect consumption, and the potential for using insects as an alternative source of income generation. Focus groups consisted of 11 to 15 participants per session, with each session lasting 45–60 min.

### 2.3 Data analysis

Quantitative data collected include demographic information on each focus group participant consisting of age; years in the community; relationship status; number of children, if they have used an MWH themselves; their role in the community; and the county in Liberia where they reside. Descriptive statistics including frequencies and means were conducted in SPSS v24.0 (Armonk, NY).

Qualitative data were analysed using the constant comparative method described by Glaser (1965, 1992) and guided by the focus group questions. Each focus group discussion was audio recorded and transcribed verbatim. The transcripts were translated from the local language into English by an independent translation service in Liberia and typed into an electronic copy. Transcripts were then read and reread by the study team to identify and label general codes. Initially, two RAs and one researcher analysed the data separately and then met together to debrief and record observations and impressions while in the field. Data were coded and then assigned to categories that clustered together by the field team and two additional researchers. (Supporting Info, Code Sheet). Finally, common themes and subthemes were developed to explain the data and discussed until agreement was reached. Each primary theme was developed from subthemes, which provided more specific, in‐depth understanding of the topic. The four primary themes and eight subthemes that emerged paint a picture that will help to inform the viability of using insect consumption as an approach to combating food insecurity within MWHs.

### 2.4 Ethical considerations

Institutional review board approval was received from the University of Michigan Health Sciences and Behavioral Sciences Institutional Review Board and the University of Liberia Pacific Institute for Research and Evaluation.

## 3 RESULTS

Research assistants conducted 18 focus groups throughout 9 of the 15 counties in Liberia. Focus group participants represent a mix of chiefs and community leaders, women of reproductive age, TBAs, women currently staying at MWHs, and male partners of these women. The majority of focus group members had utilised MWHs in the past or knew someone that had utilised an MWH. Select demographic data describing the 255 participants are presented in Table 1.

**Table 1:** Demographic information of focus group participants

Demographic characteristic	Number of participants	%
	*n* = 255	
Age^a^		
*M* (*SD*)	40.28 (15.58)	
Years in communitya		
*M* (*SD*)	27.15 (19.60)	
Relationship statusa		
Married	177	70.5
Divorced/separated	25	9.9
Widowed	5	2.0
Single	43	17.1
Unanswered	1	0.4
Number of childrena		
*M* (*SD*)	4.80 (2.95)	
Has used MWHa		
Yes	146	58.2
No	103	41.0
Unanswered	2	0.8
Role		
Chief	16	6.3
Community leader	32	12.5
Woman of reproductive age	76	29.8
Traditional birth attendant	42	16.5
Woman at MWH	47	18.4
Male partner	42	16.5
County		
Bomi	15	5.9
Gbarplou	15	5.9
Grand Bassa	24	9.4
Grand Cape Mount	15	5.9
Lofa	41	16.1
Margibi	29	11.4
Montserrado	12	4.7
Nimba	44	17.3
Rivercess	60	23.5

*Note.* Four participants did not answer.

Abbreviation: MWH, maternity waiting home.

a *n* = 251.

**Figure 1:**
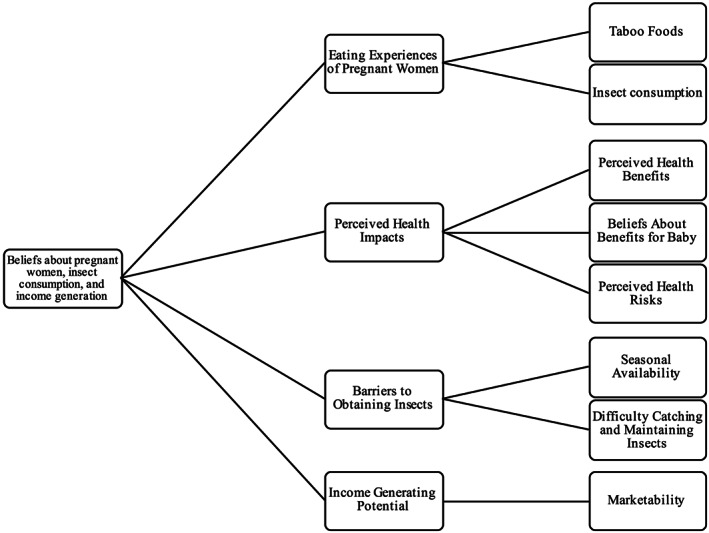
Conceptual framework—overarching concept, primary themes, and relationship to identified subthemes

Analysis of focus group discussions revealed four primary themes and eight subthemes, which were supported through comments from various focus group participants. The primary themes included (a) eating experiences of pregnant women, (b) perceived health impacts of insect consumption, (c) barriers to obtaining insects, and (d) income‐generating potential of insects. These themes and their corresponding subthemes inform us of Liberian beliefs about pregnant women, insect consumption practices, and the potential for income generation with certain species of edible insects (see Figure 1).

### 3.1 Eating experiences of pregnant women

Eating experiences of pregnant women is the first primary theme. It is supported by the subthemes (a) taboo foods and (b) insect consumption.

Focus group participants were asked to describe foods that are considered to be *taboo*, which women are encouraged to avoid during pregnancy. One of the most commonly mentioned items to avoid during pregnancy is alcohol. However, the participants listed very vague reasons, simply giving general responses such as “it can affect them and their baby” or “it will cause problems.” Although this is a commonly accepted taboo, there are other foods that are believed to cause very specific negative effects for pregnant women. One example is that pregnant women are encouraged to avoid dry foods, which is predominantly rice without any oil. Such dry food is believed to lead to a variety of problems, such as a “shortage in their blood supply” for the woman and the “child being malnourished.” Pregnant women are also told to not eat cassava snake meat “because they believe that the child will fold as the snake does.” Another focus group participant gives the rationale why women should not eat cassava snake meat when pregnant, saying, “the reason why they said that we should not eat cassava snake is because it is slow in movement and it is also stubborn in attitude.” This belief is demonstrative of Liberian cultural perspectives on the relationships between the characteristics of the foods they eat and the effects on the consumer.

In addition to these taboos, participants explained their own *insect consumption experiences*, particularly as they pertain to pregnant women. No insects were included among the foods that focus group participants considered to be taboo for pregnant women. In fact, participants highlighted that all people consume insects and pregnant women are some of the primary consumers of edible insects.

One of the most commonly listed insects that is considered edible was the “bugabug,” which refers to the termite. One participant declared, “when it comes to [termite] time, no children or big person business inside, all of us can eat it.” Another commonly mentioned insect is the “bamboo worm,” which is a term that Liberian natives use interchangeably to refer to the larvae of two separate species of insects that lay eggs in palm wine trees. One participant distinguishes the two species and describes the process for acquiring them by saying:

As for the bamboo worm, in my rubber bush the palm tree that was there I kill some of them. So in that place the [adult palm weevil] can lay egg there. While it laying egg there myself can catch it. I bring it too. But that egg will survive then the bamboo worm will go inside then I can get it out and bring too for us to eat. The difference between the palm worm and the bamboo worm is that the bamboo worm is more greasy than the palm worm. The bamboo worm does not have much [stool] while the palm worm has much [stool] in it.

Aside from this distinction, participants describe the bamboo worm in a way that suggests they are referring to both the bamboo worm and palm weevil larvae. For the purposes of analysis, we will refer to both species collectively using the term palm weevil.

Other edible insects that participants discussed include crickets, grasshoppers, and a species of insects found in dying rubber trees, which is referred to as *rubber disease.* The beliefs shared through these focus group discussions demonstrate that pregnant women are not excluded from insect consumption in Liberia and, in fact, are some of the main consumers of insects. Each of the species listed contributes to the unique and variable diets of people throughout Liberia.

### 3.2 Perceived health impacts

The second primary theme is the perceived health impact of insect consumption, and it describes the many convictions participants hold about the health impacts of the foods they consume. This holds true for the various species of insects that are consumed in Liberia as well. The second primary theme is supported by the subthemes of (a) perceived health benefits, (b) beliefs about benefits for baby, and (c) perceived health risks.

Most of these beliefs reflect positive attitudes towards insect consumption, such as *perceived health benefits* for all consumers and *beliefs about benefits for the baby* when pregnant women eat certain species. In general, focus group participants felt that edible insects are a beneficial food source because they “[keep] you healthy.” For pregnant women in particular, insect consumption is becoming a more recognised and acceptable practice. One woman stated:

The [head TBA] told me that it is good when you are pregnant to eat rubber disease and bamboo worm. I said I have never had that one, she said go ask to the hospital. When I came [my nurse] tell me the same thing so I start to eat it. The reason of me eating it is because I was educated on it, that it will give me more vitamins, and also the child will be healthy in the body.

This statement demonstrates that pregnant women are receptive to advice and education regarding the practice of insect consumption during pregnancy, both from TBAs and from healthcare workers at the hospital. Another statement made about the palm weevil larva in particular is that it can give the baby “ringer neck.” Many focus group participants mentioned “ringer neck” as a positive benefit for the mother and child. “Ringer neck” refers to a baby receiving sufficient nutrition, in the sense that the skin and fat on the back of their neck appears as “rings.”

Study participants did address some concerns about *perceived health risks* associated with insect consumption, namely, the risk for diarrhoea. This negative effect is mostly considered a problem of the insects being prepared improperly but can also result from eating too much of the insect at one time. Participants described this by saying, “there is some problem when it comes to the preparation” and “if you eat [the insect] with greediness, it can give you stomach running.” However, many focus group participants also insisted, “nobody can be sick from eating [insects].” These concerns highlight the need for careful consideration regarding preparation and species selection when determining edible insects for consumption.

### 3.3 Barriers to obtaining insects

There are additional logistical concerns that can arise when obtaining insects. Barriers to obtaining insects is the third primary theme derived from the focus group discussions and includes the subthemes (a) seasonal availability and (b) difficulty of catching and maintaining edible insects.

First, each insect has particular *seasonal availability.* Multiple focus group participants stated, “the palm worm has no special season,” suggesting that it is one of the edible insect species that is available year‐round in Liberia. Conversely, one focus group participant explains, “the [termite] can come per season and we can't buy them.” Many other participants echoed this concern of the seasonal availability of the termite, stating that it is predominantly obtainable in the dry season. The grasshopper was also noted to emerge during the dry season, whereas the “rubber disease” is said to be available any time.

Another potential problem to consider is the *difficulty of catching and maintaining edible insects.* One focus group member explained the challenges of acquiring live insects by stating, “it sometimes escape[s] from us when we try to catch them unless we cut down the whole bush to make it easy for us to catch them.”

Hunting for the termite requires particular patience and careful technique. Focus group participants described how termites build a mound where they hole up and grow and then they come outside when it rains or sometimes at night. When a termite is in the mound, it is extremely difficult to catch, but when the termites emerge, they are considered to be much easier to acquire.

Hunting for palm weevil larvae in the palm wine tree also requires patience. Once the adult palm weevil lays eggs, they hatch, and the new larvae will start to consume the tree, which creates evidence of the tree rotting. It is while the palm weevil is in this larvae stage that Liberians prefer to consume the insect. One participant explains:

You have to go hunt for [the palm weevil larvae] in the bush by checking on the palm wine trees for look for the signs that can indicate that they are there before you can cut down the tree to get it out and other people don't have the patience for that.

The grasshopper and the “rubber disease” are two additional insects that are commonly acquired for consumption. Grasshoppers are easier to catch when they are available, and they are often cleaned and fried for eating. The “rubber disease” is another species that is acquired from trees, particularly the rubber trees in the many rubber farms that exist throughout Liberia. As a focus group participant states “when it enters the tree it makes sure that the tree dies … then the disease enters, and the disease will grow inside.” It is then fairly simple to hunt the insects from the rotting trees; however, this particular species is only readily accessible to those with rubber farms in walkable distance.

### 3.4 Income‐generating potential

The fourth and final primary theme derived from the data is income‐generating potential. It is supported by the subtheme of marketability and informs the feasibility of commercially farming and harvesting edible insects for profit.

Perspectives varied among focus group participants regarding the *marketability* of various species of edible insects. It is not uncommon for people to sell the insects they have collected themselves when they have more than enough, and conversely, they can purchase them from vendors if they so desire.

The one species that was singled out as being unmarketable in some cases is the termite. The participant who made this statement did not give an explanation but simply remarked, “for the [termite], it is not marketable.” Nevertheless, a participant from a separate focus group listed the termite as one of the species of edible insects that can be bought at market but said that it costs a lot less than the other species, so it would generate less money.

Alternatively, the palm weevil was noted to be very marketable by multiple focus group participants. One woman said that if her husband goes out and gets plenty of it, she will save some for herself and cook it with rice, and then, she will sell the remaining insects to get more food.

A few focus group participants also described the marketability of the grasshopper and the “rubber disease.” One woman described hunting grasshoppers with her children and gathering some for themselves and then selling the rest for money. She regarded this as a common practice in Liberia. Another participant considered the “rubber disease” to be a species that people can hunt for and sell to make significant money. He stated, “I heard a story from Firestone area, that a fellow sold rubber disease until he was able to build house. When they are in abundance you are able to make money.” This success story of insect hunting and selling reflects the potential for income generation with certain species of edible insects, particularly when they are readily available in the area.

## 4 DISCUSSION

Our findings indicate that, across a wide variety of focus group members, insect consumption is viewed as an acceptable and even common form of dietary supplementation. Despite noting many different foods and animals that participants believed to be taboo for pregnant women, none of the focus group members listed insects among the foods they considered to be harmful for pregnant women to consume.

Given the need for improved food security at MWHs (Chibuye et al., 2018), it is crucial to examine alternate methods of dietary supplementation and income generation for women staying at MWHs to increase the use of MWHs in Liberia. Across Africa, cultural and personal beliefs about the effects of food on the unborn child dictate what pregnant women eat and what they avoid consuming during pregnancy (Arzoaquoi et al., 2015; Lennox, Petrucka, & Bassendowski, 2017). Contrary to a previous study in Tanzania, where the longhorn grasshopper was considered to be taboo for women and children (Mmari et al., 2017), focus group members in this study did not name any insects among the foods they consider to be taboo for pregnant women. In fact, they endorsed beliefs about insect consumption that showed benefits for the unborn fetus, such as sufficient nutrition to give the baby “ringer neck.”

Participants described the logistical barriers and considerations when attempting to obtain, contain, and prepare insects. These difficulties may include seasonal availability, troubles finding and capturing live insects, and underprepared insects causing sickness. However, participants also described instances in which they themselves have hunted for palm weevil larvae, which they could then eat and sell for monetary gain. Income‐generating activities such as this are an important means of providing both sustenance and monetary gain. Muafor et al. (2015) demonstrate that a grub farming system of raising palm weevils in boxes can help to produce a contained source of pam weevil larvae. This approach has the potential to address some of the logistical barriers brought up in focus group discussions.

Based on the responses from focus group participants, the example set by others and advice given by TBAs and healthcare providers is a significant source of encouragement for the practice of insect consumption and often dictates which species are considered acceptable to consume. A woman's decisions during pregnancy and delivery depend heavily on a complex interplay between personal preferences and input from TBAs (Greatrex‐White & Monaghan, 2014). Beliefs regarding the positive impact of insect consumption on the physical characteristics of an unborn child offer motivation to seek out and consume various species when pregnant, particularly the “rubber disease” and the palm weevil larvae. Focus group participants also noted that women play a central role in the acquisition and preparation of edible insects for their families. In the same way, it is important to include the pregnant women at MWHs and input from TBAs in the future implementation of an insect harvesting intervention at MWHs.

Throughout the focus group discussions, participants corroborate and expand upon the foundational research that establishes palm weevil larvae as an excellent means of implementing a nutritious complimentary food source that can also be sold for profit (Laar et al., 2017; Van Itterbeeck & Van Huis, 2012). Focus group members noted the year‐round availability and higher marketability of the palm weevil larvae, which would make them an ideal species to utilise as an alternate source of nutrition for MWHs in Liberia. Additional research is needed to test the outcome of utilising this approach in MWHs. Results from this study regarding the success of gathering and selling insects on a small scale provide a platform for the application of this method in MWHs.

### 4.1 Limitations

One limitation to this study may be the potential power differential between some of the focus group participants, as focus group participants included both men and women and chiefs and community leaders as well as general community members. Additionally, due to slight differences between Liberian English and American English, there may be meanings behind phrases that are not fully recognised by American researchers. Although there are limitations to this study, we believe that the research reported here has several key strengths. The study includes a diversity of perspectives including chiefs, community leaders, women of reproductive age, TBAs, women currently staying at MWHs, and male partners from 60% of counties in Liberia. It includes participants from a number of different tribes within Liberia and reflects a wide view from multiple perspectives. This comprehensive approach strengthens our findings and presents important baseline data to build on for future research.

## 5 CONCLUSION

This study demonstrates that insect consumption is a common and acceptable practice for participants from across Liberia. Furthermore, participants highlighted that, despite cultural taboos regarding certain foods, pregnant women are not excluded from insect consumption and, in fact, are even encouraged to seek out certain species to promote desirable qualities in the unborn fetus. Focus group analysis has also affirmed that participants consider insect farming to be a source of dietary supplementation and a feasible method of income generation. Further research is then needed to determine the effectiveness of this approach in promoting a sustainable source of nutrition and income for women who utilise MWHs in Liberia.

## CONFLICTS OF INTEREST

The authors declare that they have no competing interests.

## CONTRIBUTIONS

JRL, KMC, JEP, CAM, and JPA conceived the study. JRL, JEP, and CAM developed and designed the study. JEP, AN, and AK were responsible for data acquisition. KMC, JEP, AN, and JRL were responsible for statistical data analysis and interpretation of the data. All authors helped to draft and revise the manuscript. All authors read and approved the final version.

## Supporting information

Supporting info item

Supporting info item
