# Supplementary material for: Acceptability and feasibility of insect consumption among pregnant women in Liberia
Source: Matern Child Nutr. 2020 Mar 1;16(3):e12990. doi: 10.1111/mcn.12990 (PMC7296793; doi:10.1111/mcn.12990)
Supplement: Supplementary file 1 — Supporting info item [file MCN-16-e12990-s001.docx]

**Original Codes Grouped by Theme**

- Theme 1: Getting Edible Insects
  - Species
  - Season
  - Hunt
  - Buy
  - Challenges
- Theme 2: Consumers
  - Pregnant Women
  - Children <5
  - Babies
- Theme 3: Pregnant Women
  - Main Food
  - “Taboos”
  - Supplies (brought to MWH)
- Theme 4: Income Generation
  - Yes/no
- Theme 5: Benefits to Insect Consumption
  - Benefits
- Theme 6: Health Risks with Insect Consumption
  - Health Risks

**Final Themes Developed from Codes**

- Main Theme 1: Eating Experiences of Pregnant Women
  - Main Foods
  - Taboo foods
  - Insect consumption
- Main Theme 2: Perceived Health Impacts
  - Perceived Health Benefits
  - Beliefs About Benefits for Baby
  - Perceived Health Risks
- Main Theme 3: Barriers to Obtaining Insects
  - Seasonal Availability
  - Difficulty Catching and maintaining insects
- Main Theme 4: Income Generating Potential
  - Marketability
