# Supplementary material for: Acceptability and feasibility of insect consumption among pregnant women in Liberia
Source: Matern Child Nutr. 2020 Mar 1;16(3):e12990. doi: 10.1111/mcn.12990 (PMC7296793; doi:10.1111/mcn.12990)
Supplement: Supplementary file 2 — Supporting info item [file MCN-16-e12990-s002.docx]

**Focus Group Guide for Community Members**

Community Name: _____________________

County: ___________________

Facility Name: _________________________

Type of community member and number of attendees:

- Chief________
- Community Leader_________
- Women of reproductive age___________
- TBA/TMs_________
- Women currently staying at MWHs___________
- Male partners_____________

Thank you for taking the time to meet with us today. We are interested in learning more about maternity waiting homes in Liberia and how they are working here.

1. Maternity Waiting Home Questions
2. Do you know if community members contribute to supporting the ongoing costs of maternity waiting homes here? Yes / No / Don’t know
   1. Can you tell me more about that? (probe: what type of support, how much, how does it work)
3. Are community members involved in the governance of MWHs? Yes / No / Don’t know
   1. Can you tell me more about that? (probe: how are maternity waiting homes governed? how are community members involved? How many community members? How are they selected? Etc.)
4. Do you have a formal role in the governance of the Maternity Waiting Home? Yes / No If yes, what is your role?
5. Were you involved in the design, construction, or maintenance of the MWH in your community? Yes / No
   1. Can you tell me more about that? (probe: who was involved in the design and construction of the MWH? Who is involved in the maintenance?)
6. Do you think the maternity waiting home affects how you or others in the community think about the health facility?
7. What do you think is good about having a Maternity Waiting Home in the community?
8. (If female), did you use a MWH for your last delivery? Yes / No
   1. May I ask, why / why not?
   2. If Yes: Who decided, you should stay at the MWH? Why?
   3. What did you bring with you?
9. How did you first hear about the Maternity Waiting Home?
10. How has awareness of the MWH spread in this community? (probe: Do you think most people know about it? Why / why not?)
11. What do you think is the ideal amount of time for women to stay at a maternity waiting home before delivery and after delivery?
12. What are things you think that MWHs are doing well?
13. What are things you think that MWHs could do better?
14. What are things that you think might prevent women from using MWHs?
15. Finally, are there ways that you and your community support the MWH?
16. Food Security and Procurement Questions
17. How do you think the community can help in the provision of food for the MWH?
18. What types of food is most common for pregnant women to eat in your community?
19. Are there any foods that pregnant women do not eat or are not supposed to consume? Why not?
20. What food do women normally bring to the maternity waiting home with them?
21. Do community members eat insects or bugs in your community?
    1. If yes, what kinds of insects and bugs are consumed? (examples: bug, beetle, termite, ant, bee, caterpillar, grasshopper, oil palm weevil, shea nut weevil, fly, cricket, etc.)
    2. Who are the main consumers? (children, adults, male, female)
    3. Which metamorphic (Developmental) stage is consumed? (examples: Egg, Larvae, Pupae, Nymph, Imago/adult)
    4. How do you get these insects? (hunting them yourself, buying them)
       1. If you buy them, how much do you pay for them?
       2. If you hunt them, how far do you go? What difficulties do you encounter?
22. When are these insects available? (Before rainy season, during rainy season, after rainy season)
23. Do pregnant women eat insects or bugs here in your community? Why or why not?
24. Do young newborns eat insects or bugs here in your community? Why or why not?
25. Do children under the age of five eat insects of bugs here in your community? Why or why not?
26. Do you see the potential of edible insects as an alternative source of generating income? If Yes, how?
27. Are there any challenge faced in eating these insects? If yes, what are the challenges?
28. Does eating of these insects pose any health risk?
    1. If yes, what type of health risk/kind disease is encountered?
    2. Are pesticides (chemicals/insecticides) used on the crops or land the insects are harvested from?
       1. If yes, do you know what kind of pesticides (chemicals/insecticides) are used?
